# Psychosocial, behavioural, pedagogical, and nutritional proposals about how to encourage eating a healthy breakfast

**DOI:** 10.1186/s13052-014-0073-7

**Published:** 2014-08-13

**Authors:** Chiara Mameli, Erica Galli, Dario Dilillo, Alberto Alemanno, Loredana Catalani, Silvia Cau, Lucia Fransos, Fabio Lucidi, Agostino Macrì, Paolo Marconi, Alessandro Mostaccio, Giovambattista Presti, Giuseppe Rovera, Giuseppe Rotilio, Mariagrazia Rubeo, Carla Tisiot, Gianvincenzo Zuccotti

**Affiliations:** 1 Department of Paediatrics, University of Milan, “Luigi Sacco” Hospital, Milan, Italy; 2 École des Hautes Etudes Commerciales de Paris, Paris, France; 3 Association of Italian Pedagogists (ANPE), Rome, Italy; 4 Istituto Universitario di Lingue Moderne - IULM University of Milan, Milan, Italy; 5 Italian Association of Food Science Specialists (ANSiSA), Milan, Italy; 6 Department of Psychology of Developmental and Socialisation Processes, University “La Sapienza” of Rome, Rome, Italy; 7 National Consumers Union (UNC), Rome, Italy; 8 Movimento Consumatori, Rome, Italy; 9 Department of Human and Social Science, Kore University of Enna, Enna, Italy; 10 San Raffaele University of Rome, Rome, Italy; 11 Italian Eating and Weight Disorders Association (AIDAP), Rome, Italy

**Keywords:** Breakfast, School, Children, Behaviour analysis, Nudging, Cognitive strategies, Games

## Abstract

**Background:**

Even if more and more evidences have highlighted the importance of breakfast in the growth and development of children, from 10 to 30% of US and European children and adolescents regularly skip breakfast. Thus, there is still a lot to be done before breakfast becomes a daily habit. The aim of this paper is to try and understand how it is possible to overcome the real or imaginary difficulties associated with skipping breakfast by psychosocial, behavioural, pedagogical and nutritional proposals.

**Discussion:**

Schools are the best context where perform healthy interventions because it is here that children learn about the importance of good health at an age when the school still plays a major role in their education. Some school interventions, based on solid theories as the Self Determination Theory and the Behaviour Analysis, have been implemented in the last years to promote health behaviour such as intake of fruit and vegetables and physical activities. Cognitive behaviour therapy is the most closely monitored type of treatment/cure for obesity in randomised controlled trials. Moreover some associations such as the National Association of Food Science Specialists have drawn an own method to encourage food education at school and promote the importance of prevention. These projects could be used as starting point to perform interventions focus on breakfast.

**Summary:**

Increase the consumption of breakfast between children is very important. Efforts should be done to drawn new school projects based on scientific-evidences.

## Background

In the last years more and more evidences have highlighted the importance of a healthy diet in the growth and development of children. Breakfast is a strategic daily meal that plays an essential role in ensuring the good health and wellbeing of an individual [1]. It is associated with healthier macro- and micronutrient intakes, body mass index and lifestyle. Moreover breakfast improves cognitive function, intuitive perception and academic performance [1]. Nevertheless, from 10 to 30% of US and European children and adolescents regularly skip breakfast, with a higher percentage among adolescents and the female population [2]. Thus, there is still a lot to be done before breakfast becomes a daily habit. The aim of this paper is to try and understand how it is possible to overcome the real or imaginary difficulties associated with skipping breakfast by psychosocial, behavioural, pedagogical and nutritional proposals.

## Discussion

### Choosing to change: lessons to be learnt from healthy lifestyle projects in primary schools

In the last decades many healthy interventions have been performed in schools because it is here that children learn about the importance of good health at an age when the school still plays a major role in their education. Data shows that primary school children already think (and possibly worry) about their weight and appearance [3],[4]. Furthermore, the lifestyles and dietary behaviour developed as a child tend to consolidate during adolescence. This is why many interventions are implemented in primary schools; they focus on children who are at an age when problems such as overweight and obesity tend to develop and consolidate, but before puberty when these problems become even more difficult to tackle.

In the last three decades interventions to promote healthy behaviour at school have undergone a cultural shift. They were initially intended to “teach” children, adolescents, and/or their parents about the importance of healthy lifestyles and associated behaviour by face-to-face lessons held by experts mostly from the health sector. These lessons were later shown to be ineffectual because they failed to encourage the development of the tools needed by children and adults to resist the pressure of unhealthy lifestyles [5],[6]. Afterwards, a cultural shift encouraged by the World Health Organisation (WHO) through the Alma Ata Declaration [7] and the “Ottawa Charter” happened [8]. These two WHO documents aired the idea that health is a personal and social responsibility and emphasise the importance of tangible social policies involving the active participation of citizens. Consistent with these principles more recent interventions were no longer based on a purely informative paradigm, but on the reinforcement of skills and a correct understanding of an individual’s psychophysical, behavioural, emotional, relational and social characteristics, enabling a person to see himself as an active subject responsible for his own behaviour (and health) in a social context.

Han *et al.* have recently published a review of literature on childhood obesity in which they drew attention to some very interesting issue about preventing interventions [9]. Firstly they highlighted as preconceived ideas rather than an in-depth review of literature are often the basis of the scientific, political, and cultural debate about which basic characteristics provide the most successful interventions to positively modify unhealthy nutritional behaviour. Secondly they reported the fact that, although many interventions were successful, they were not repeated or regularly planned because they lacked solid theories explaining how the desired behavioural changes had been induced. The third issue underscored by Han *et al.* is the risk that good dietary behaviour interventions might ultimately heighten the stigma and social stereotypes associated with overweight and obesity and in the end worsen the body image and social adaptation of children and adolescents considered “fat”. These theoretical concepts have inspired the operational strategies of several school intervention programmes, such as the project entitled “Diamoci una mossa” (Let’s get moving) launched for the first time in 2006 by the Unione Sport per Tutti in Italy [10],[11]. More than 6,000 classes and 130,000 children and their families have been involved in this project, based on the theory that physical exercise and a healthy diet are complementary elements in the right to health. It is an example of nationwide intervention based on precise theoretical model, the Self Determination Theory (SDT) [12]. The SDT studies the processes of self-regulation of human motivation within social environments and considers the self determination as an innate psychological need: the need for autonomy, competence, and relatedness. In line with the self-determination theory, trained operators first proposed healthy behaviours (related to food and physical activities) as games to children and their families. Then, to try and make the children feel more independent, they were asked to build their own games; this was intended to encourage appropriate and salutogenic physical and food behaviour. Moreover schools were involved in a self-monitoring process (by diary) to internalise healthy behaviour. Approximately 2,000 children were chosen from amongst all the participants using a random sampling procedure to assess the efficacy of this intervention. The assessment procedure entailed the evaluation of BMI and weekly physical activity level by the “International Physical Activity Questionnaire” before the start of the project and at the end [13]. The results of analyses show that BMI increased in underweight children and decreased in overweight children at the end of the intervention and a significant reduction in the number of minutes/week during which the children are involved in sedentary activities [p < .001] - a drop from an average of 372 at the outset to 335 at the end of the project - and an increase in the time spent doing moderate or vigorous activities – an increase from 219 before the start to 255 at the end of the project [14].

As “Let’s get moving”, we need science-based initiatives for the future, especially in primary schools, to promote an active lifestyle and a healthy diet.

### Strategies and programmes to improve children’s eating habits: a behavioural study

There are two ways in which to analyse human behaviour, including eating: form (how it is presented) and function (which environmental variables will induce eating and maintenance). The focus of Behaviour Analysis, a branch of psychology developed 80 years ago, is to identity this/these independent (manipulable) variable/variables where behaviour (dependent variable) is the function. Like any other behaviour, learning, be it functional or dysfunctional, determines what we eat and why we prefer certain foods and not others. According to Azman, postnatal feeding and the ensuing transition phase to an adult diet during the child’s first year of life is the period during which the parent can help to establish good eating behaviour by using the “right” strategies, and reduce the likelihood that the child adopt problematic food behaviour [15]. Parents not only share their genes with their children (and therefore possible predisposition), they directly influence their offspring’s food behaviour and give rise to what is defined in literature as an obesogenic environment. Birch and Fisher have identified several factors influencing children’s preferences and the consumption of certain foods [16]:

 early experience with a wide variety of flavours (starting with mother’s milk);

 repeated opportunities to sample new foods (food has to be eaten 5 to 10 times to increase consumption of that foodstuff); this is one way to battle neophobia, the refusal to taste new foods;

 preference for familiar foods, i.e., present in the environment.

A child also develops his own preferences and behaviour by observing role models such as his parents, other adults, friends, and siblings. In this case learning has been shown to be successful when the model is similar to the observer, and is even more effective when the model is a child slightly older than the observer [17]. These considerations are valid not only for behavioural excesses such as eating too much or too quickly, but also for behavioural *deficits* such as refusing to eat certain basic foods, such as fruit and vegetables, or skipping meals, something that often happens at breakfast. The food problems and food rejection which occur at breakfast are particularly interesting because they are linked to obesity. For example, out of 1,202 children in a sample in northern Italy, 22% said they skip breakfast; 27.5% were overweight and 9.6% obese, against 9.1% and 4.5% respectively of the remaining 78% of children who said they ate breakfast [18]. The *Applied Behaviour Analysis* (ABA) is defined as the science that applies the principles of behaviour to improve socially significant behaviour, and in which experimentation is used to identify the variables responsible for behavioural changes. The *Food Dudes Behaviour Change Programme for Healthy Eating* is a food education programme based on Behaviour Analysis, set up by the Department of Psychology at Bangor University (Wales-UK) to encourage and teach children to adopt suitable and healthy dietary behaviour (consumption of fruit and vegetables). *Food Dudes* was used in schools [19]. Several international studies confirmed its efficacy, and in fact one version was developed for children ages 4–11 [20]-[22], and another version for children ages 2–4 [23]. The programme is based on three well-known principles of experimental psychology applied to education [24]:

*repeated tasting*. Children are repeatedly fed small portions of fruit and vegetables. The success of this method has been demonstrated by studies showing that the more the child tastes a food, the greater the chance he will learn to like it;

*role modelling*. Children watch exciting movie adventures featuring the *food dudes* fighting against a band of evildoers who want to rob the planet of its life-giving force, i.e., fruit and vegetables. Children are encouraged to taste these foods to help in the fight, and receive rewards;

*rewards*. If they taste and eat the portion of fruit and vegetables they are rewarded with gadgets they can use at school (e.g., colour pencils, flasks, pedometers). This positive reinforcement principle makes children more likely to taste the food in the future; this also depends on the fact that the teacher eats the same food (fruit and vegetables) given to the students.

In Italy the programme has been used in two tests (with a control group) in two different geographical areas (Sicily and Lombardy). In the schools in Milan, not only was there an increase in the consumption of fruit and vegetables during the short period the programme was implemented, but statistically significant changes were noted in the calorie intake (*sugars, fibres, sodium*) of children who brought their snacks from home. The study in Sicily demonstrated that *Food Dudes* worked successfully with obese children who ate the same quantity of fruit and vegetables as their normal weight peers; this did not occur in the control group [25].

An *ad hoc Food Dudes* programme could be elaborated to encourage children to eat breakfast. The parents should play a strategic role and should not use coercive methods to make children eat in the morning; they should be a behavioural model for their children, also at breakfast. A parent who skips breakfast and expects to be able to force the child to eat in the morning is not using a successful strategy. Children should be offered a wide variety of foods to eat during their first meal of the day and be recompensed with food “*reinforcers*” (rewards).

### *Nudging*: the “soft prod” towards good habits – the role of behavioural research in public health initiatives

In the last years Governments have implemented the use of behavioural science [26] and *nudging*[27] to encourage people to adopt healthy lifestyles without limiting their freedom of choice. This approach, inspired by “libertarian paternalism” [28], assumes that the goal of public policies should be to help citizens make better choices for themselves and society, and yet leave them free to make their own choices [29],[30]. As architects of this choice, Governments establish the context, process, and environment in which citizens make that decision. In particular, this kind of approach appears well-suited to prompt, almost *guide* people to adopt preventive health behaviour. In fact, when we talk about lifestyle we are actually talking about habits; as a result, *nudging*, rather than top-down rules, may be successful. A healthy, regulated diet may be compatible with this approach, because people tend to commit to something with short-term results and immediate rewards. It is really difficult for people to commit to changing their habits to avoid becoming ill or having other health problems later on in life, and the depressing effect *memento mori* of many (useless) information campaigns has not helped at all. On the contrary, focusing on small, rewarding, and immediate changes could be the right recipe: *nudging* cannot ignore the challenge of how to encourage rather than coerce people to eat regular breakfasts. For example to fight laziness we could use some strategies such as a breakfast box, *i.e.,* ready-to-eat yogurt packs/milk, orange juice/fruit, cereal portions/biscuits. The consumer could choose his own combination, for example after reading a brochure describing different kinds of breakfasts and helping him make a nutritionally healthy breakfast based on his own likes and dislikes. Obviously these *boxes* should be widely distributed and easily accessed rather than niche products. Moreover it could be institutionalize breakfast at school or at work to the purpose to use peer pressure and society to persuade people to take the time to have breakfast.

### The theory behind cognitive behavioural therapy to treat obesity: cognitive-behavioural strategies to encourage a healthy breakfast

Cognitive Behavioural Therapy (CBT) provides obese individuals with a set of principles and techniques to change their dietary habits and encourage an active lifestyle and regular physical activity. The idea began to circulate in the sixties in the United States, and is the most closely monitored type of treatment/cure for obesity in randomised controlled trials [31]-[33]. CBT was initially developed based on the premise that learning principles could be a valid tool to correct the dysfunctional behaviour patterns of individuals with weight problems [34],[35]. However, after their initial enthusiasm scientists agreed that factors such as genetic predisposition and the biological mechanisms (as cognitive processes) associated with obesity hindered weight loss maintenance and the behaviour acquired during therapy. Some studies have underscored the need to couple the CBT programme with strategies aimed at producing suitable cognitive changes and encouraging achievement of the obesity treatment goals, *i.e.,* weight loss and weight loss maintenance. Obesity applied CBT focuses on education and involves not only teaching a patient how to eat, but also encouraging him to have an active lifestyle; this will make him aware of the biological, psychological and environmental obstacles to weight loss and weight maintenance; the aim of the programme is to make him his own *therapist*. In fact the therapist does not teach the patient how to solve a particular problem, instead he asks him questions with open answers to encourage the patient to find the most appropriate solutions (as part of a Socratic dialogue). Treatment is generally divided into two stages: at the beginning the goal is to loose weight and tackle the obstacles preventing weight loss and weight loss maintenance, then, after 5–6 months, the patient moves to the second stage where the goal is to help patients assume the approach and behaviour best suited to maintaining the results they have achieved. The tools include: self-monitoring of food intake (counting calories), physical exercise (with a pedometer), and weight (weight log). Moreover treatment includes a procedure to identify and discuss possible dysfunctional thoughts, which might induce the patient to drop out of the weight loss programme. In particular during the first sessions, the patient is asked to keep a “Diary of the obstacles to weight loss” to help him understand events, thoughts, emotions and behaviour, and *restructure* the thoughts which cause him to overeat. Scientific research on *lifestyle modification* to treat obesity has produced positive results as showed by Wadden *et al.*[36]. CBT could be used to make breakfast a daily habit. The reasons why people skip breakfast could be examined using a questionnaire highlighting the obstacles which occurred “often” or “always” (*i.e*., lack of time or appetite in the morning), and for which specific solutions could be provided. Strategies to overcome these obstacles could include, for example, education, and mechanical feeding to re-establish normal signs of hunger or fullness or even so-called problem solving. Education can help increase awareness about the importance of breakfast, the prevention of weight problems and insulin resistance, and the improvement of cognitive performance. The best solution is a limited number of short and simple messages encouraging and explaining the importance of breakfast as a way to regulate calorie intake during the day. Mechanical feeding could be one way to solve the problem of appetite loss in the morning (often cited by individuals who skip breakfast). The cognitive behavioural strategy consists in asking the patient to “mechanically” plan his meals ahead of time, either by himself or with a therapist, as if it were a medicine; he should disregard his physiological stimuli, thoughts about food, and ambient situation. Clinically speaking this helps to normalize the often-erratic hunger and fullness mechanisms of an individual with bad eating habits: it also helps to manage his thoughts about food and to collect data about the effects of food intake on weight swings. Problem solving can also be used as a backup to mechanical feeding in order to overcome the obstacles preventing individuals from eating breakfast. This strategy primarily involves identification and specification of the problem, implementation of solutions, and finally analysis of the entire process to establish the consequences of all possible solutions.

### The ANSiSA method for a healthy diet and active lifestyle: focus on breakfast

To increase awareness about prevention, and adopt strategies to improve the health of every individual, the National Association of Food Science Specialists (A.N.S.i.S.A.) has for many years promoted a food education alliance between Scientific Societies, the Retailing and Distribution Industry, Associations and Institutions [37]. The “ANSiSA Method for a healthy diet and active lifestyle” was developed to encourage food education at school and promote the importance of prevention [38]. Drafted by a team of multidisciplinary specialists, the method is based on direct experience and several *good practice* principles in the field of food education at school. Operationally speaking, the ANSiSA method uses a didactic kit which includes several practical tools such as slides and promotional healthy diet materials to be used by the nutrition expert, a food education handbook for the teacher, educational games for the students, feedback questionnaire for the students and families, a handbook for the families, a handbook about school meals. After the preliminary stage establishing the needs of the class and the objectives, the Method then envisages the definition of strategies and decisions about the most suitable didactic materials needed to help the recipients achieve that *know-how*. To decide what to teach and how, it is also important to be aware of the learning ability of the students and their socio-cultural background: this is why the active involvement of the school/family is an important part of the food education project. The hardest, but absolutely crucial role of the teacher-tutor is to correctly motivate the students so that they adopt healthy behaviours and attitudes, not only immediately, but also in the future. It’s easier to be motivated and learn about food education and therefore improve a dietary habit, or change one’s approach towards food, if [39]:

 a person is curious, either spontaneously or after stimulation: the tutor has to use arguments and didactic tools to trigger the individual’s curiosity. These arguments and tools have to be immediately available and allow the person to see, touch, and try for himself;

 there is dissonance or contradiction: the tutor has to teach either by raising problems rather than providing answers, or by starting a lesson with a tough question which the children have to answer during class, or even by stimulating their curiosity with a quiz or a cartoon and asking the students to discover the trick or inconsistency. In this way they feel they are successful and can succeed. The tutor has to begin with easy tests tailored to the level of knowledge or ability of the pupils. He can then make them gradually more difficult and complex; before the age of six a child will normally react to direct, i.e., pleasant motivation;

 the tutor in primary schools should start by turning negative behaviour into positive behaviour;

 if the children are in a group: the tutor has to remember their strong need for affiliation; as a result, learning should be done as a group using exercises, games and group/class interaction;

 learning is followed by *reinforcement*: the tutor must provide incentives for those who change, strive, attempt and experiment, and also for students who intervene, participate and collaborate the most;

 the feedback must be immediate, frequent, explicit, direct, informative, and remedial: the tutor gives and asks for feedback to/from the class, encourages exchanges, interaction, and active participation between students.

Games can help stimulate and increase interest in the topics raised; in turn this will facilitate more and better learning. An example of game used in the ANSiSA method is *“WHAT SHALL I PUT IN MY CART TODAY?”.* This game was invented for primary school children; a more complicated version was developed for middle and high school students. The game can help to assess whether children can identity foods properly, and whether they can accurately fill the cart with the right food for a single meal, or organise a complete and balanced menu. The game also helps to stimulate their creativity and expressivity.

Rules about how to play the game:

 divide the children into two teams and give each team a cart;

 each member in each team then take turns to pick a card from the pack and decide whether to put the food illustrated on the card in the cart, or throw it away and put it at the bottom of the pack;

 when a team thinks it has all the food and ingredients it needs for a complete, balanced daily meal, it says ‘game over’;

 the teacher in his role as referee will assess the contents of the two carts and announce the winner.

Hopefully the parents will participate in several stages of the class project; some strategies to make their support more significant could be: motivate and inform parents about the advantages of a healthy diet, perform tests to establish the family’s eating habits, give the “Family Guide” and interactive work cards.

The ANSiSA Method can be adapted to focus on breakfast by reorganising the didactic tools and games used in class during general food education.

## Summary

The aim of this paper was to propose new science-based strategies to promote breakfast, especially in the schools where children are more conditioned. Class is a group where child compare oneself with others, his curiosity can be stimulated promoting the learn of health behaviour. To teach students is not enough, they have to be helped to turn information into action by pedagogical proposals including games and activities such as exploration, manipulation, communication, relationships, and psychomotor and discovery activities.

The child can be help to identify obstacles to consumption of breakfast and individual solutions should be suggested for specific problem such as mechanical feeding in case of lack of appetite in the morning. Moreover projects should involve parents as they are the first behaviour model for the child. Top-down rules should be avoid, while child and his family should be guide to choose health behaviour in a freeway.

## Abbreviations

WHO: World Health Organisation

SDT: Self determination theory

ABA: Applied behaviour analysis

CBT: Cognitive behavioural therapy

A.N.S.i.S.A.: National association of food science specialists

## Competing interests

The authors declare that they have no competing interests

## Authors’ contributions

GZ, CM, EG, DD critically revised the entire manuscript. AA, LC, SC, LF, FL, AM, PM, AM, GP, GR, GR, MR, CT drafted the manuscript. All authors read and approved the final manuscript.
